# Prevalence of Henipavirus and Rubulavirus Antibodies in Pteropid Bats, Papua New Guinea

**DOI:** 10.3201/eid1612.100879

**Published:** 2010-12

**Authors:** Andrew C. Breed, Meng Yu, Jennifer A. Barr, Gary Crameri, Claudia M. Thalmann, Lin-Fa Wang

**Affiliations:** Author affiliations: Veterinary Laboratories Agency, Addlestone, UK (A.C. Breed);; Australian Animal Health Laboratory and Australian Biosecurity Center for Emerging Infectious Diseases, Geelong, Victoria, Australia (M. Yu, J.A. Barr, G. Crameri, C.M. Thalmann, L.-F. Wang)

**Keywords:** viruses, zoonoses, Henipavirus, rubulavirus, orthoreovirus, Henda virus, Nipah virus, bats, concurrent infection, serologic surveillance, New Guinea, dispatch

## Abstract

To determine seroprevalence of viruses in bats in Papua New Guinea, we sampled 66 bats at 3 locations. We found a seroprevalence of 55% for henipavirus (Hendra or Nipah virus) and 56% for rubulavirus (Tioman or Menangle virus). Notably, 36% of bats surveyed contained antibodies to both types of viruses, indicating concurrent or consecutive infection.

The genus *Henipavirus* in the family *Paramyxoviridae* contains 2 highly lethal viruses, Hendra virus (HeV) and Nipah virus (NiV), both of which use pteropid bats as their main natural reservoir (*1*). The discovery of HeV in Australian flying foxes in 1996 (*2*) marked the beginning of a new wave of research activities, which led to the association of bats with some of the most notable viral pathogens to emerge in recent history, including NiV (*1*), severe acute respiratory syndrome–like coronaviruses (*3*), Ebola virus (*4*), and Marburg virus (*5*). In addition to the henipaviruses, 2 novel paramyxoviruses in the genus *Rubulavirus* were discovered in Australia and Malaysia. Menangle virus (MenPV) was isolated in Australia during a disease outbreak in pigs, with epidemiologic evidence suggesting the involvement of human patients as a result of pig-to-human transmission (*6*). Tioman virus (TioPV) was isolated from bat urine collected on Tioman Island in Malaysia and, although anti-TioPV antibodies were detected in residents of the island, its potential to cause disease in humans is unknown (*7*).

Several orthoreoviruses were also isolated from bats in Australia and Malaysia. Pulau virus was isolated from bat urine collected on Tioman Island in 2000; it is closely related to the Nelson Bay virus (NBV) isolated from a pteropid bat in 1968 in Australia (*8*). More recently, Melaka virus and Kampar virus, both closely related to viruses in the NBV species group, were isolated from human patients with respiratory symptoms; epidemiologic investigations strongly suggested they were the causative agents (*9**,**10*). Broome virus (BroV), a new orthoreovirus species, was isolated from a sick little red flying fox (*Pteropus scapulatus*) in 2002 in Australia, but its disease-causing potential is unknown (*11*). This study was conducted in June 2008 to survey bats in Papua New Guinea to determine the presence of various known bat viruses and to assess the potential of these viruses to be transmitted to the bat populations in Australia.

## The Study

A total of 66 bats were caught at 3 locations in Papua New Guinea (Figure; Table A1). They were anesthetized by using a combination of ketamine and medetomidine at doses similar to those stated in a previous study (*12*). Blood samples were held at room temperature for 24–48 hours and then serum separated by using centrifugation as required. Serum samples were held at 4°C until they were shipped to the Australian Animal Health Laboratory.

**Figure:**
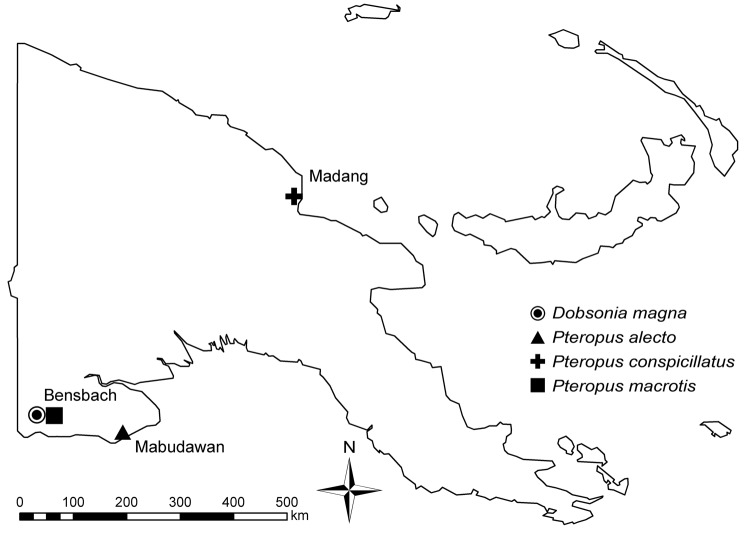
Location and species of bats collected for study of henipavirus and rubulavirus antibodies in pteropid bats, Papua New Guinea, June 2008.

Virus-specific antibodies were detected by using a variety of assays previously developed by our group at the Australian Animal Health Laboratory. For the henipaviruses, the Luminex-based binding and inhibition assays (*13*) were used for initial screening, and positive samples were confirmed by virus neutralization test (VNT). Only those with positive results in all 3 assays are shown in the Table A1. For TioPV and MenPV, initial screening was conducted by using an ELISA with purified TioPV virion as antigen. ELISA-positive samples were then confirmed by VNT against each virus. For viruses in the NBV species group, a mixture of purified recombinant sigma C proteins from NBV, Pulau virus, Melaka virus, and Kampar virus was used as ELISA antigen for initial screening. Positive samples were then confirmed by Western blot against each of the 4 recombinant proteins. For BroV, initial screening was conducted by ELISA using purified virion as antigen; positive samples were confirmed by immunofluorescent antibody test on cells infected with BroV.

A summary of the results is presented in the online Appendix Table. Seroprevalence for HeV was 50% (33/66); for NiV, 55% (36/66); TioPV, 38% (25/66); MenPV, 56% (37/66); NBV-like viruses, 17% (13/66); and BroV, 6% (4/66). The seroprevalence of the 2 types of paramyxoviruses, HeV/NiV at 55% and TioPV/MenPV at 56%, is high. The most striking finding is the presence of antibodies to both groups of viruses in 36% (24/66) of the samples. Considering that VNT is a more specific and less sensitive assay, the actual positive rate could be >36%. Compared with results of the study conducted in Madgascar (*14*), in which 1/427 serum samples contained VNT-positive antibodies to both henipavirus and TioPV, our finding suggests extremely different paramyxovirus infection dynamics in bats in Papua New Guinea. Whether these data suggest concurrent or consecutive infection is not clear. We anticipate that the use of pteropid immunoglobulin M–specific reagent (currently in development in our group) will clarify this question in the future.

The positive rate for both groups of paramyxoviruses is much higher in *P. conspicillatus* bats (spectacled flying fox) from Madang than *Dobsonia magna* bats (bare-backed fruit bat) from Bensbach. We believe this difference has more to do with the bat species than the geographic location. For henipaviruses, seroprevalence is slightly higher for NiV than for HeV, which is also consistent with the Luminex inhibition assay showing a trend of slightly higher inhibition for NiV than for HeV (data not shown). This higher seroprevalence may suggest that henipaviruses circulating in Papua New Guinea are more NiV-like than HeV-like. However, in the absence of genetic sequence data, serologic findings are inconclusive.

In addition, for the 2 rubulaviruses, seroprevalence is higher for MenPV than for TioPV. This finding is noteworthy because our previous data indicated a one-way cross-neutralization, with MenPV antibodies failing to neutralize TioPV (G. Crameri and J. Barr, unpub. data). The results from this study suggest either that the main strain of bat rubulavirus(es) circulating in bats in Papua New Guinea is more closely related to MenPV or that there are different strains circulating and the MenPV-like are more dominant.

Although the overall seroprevalence for orthoreoviruses was much lower, the results nevertheless produced some useful information. First, the prevalence of 18% (2/11) of the NBV group viruses in *D. magna* bats at Bensbach is not much different from the 20% (11/54) in *P. conspicillatus* bats at Madang, indicating the NBV group of orthoreoviruses is present in bats of different species in Papua New Guinea. Second, none of the 66 serum samples was positive for both NBV and BroV, which supports our previous conclusion that BroV is a new species in the genus *Orthoreovirus* and that no significant cross-reactivity occurs between BroV and orthoreoviruses of other species groups (*11*).

## Conclusions

In this study, a serologic survey was conducted for 4 groups of viruses, 2 from the family *Paramyxoviridae* and 2 from the genus *Orthoreovirus,* family *Reoviridae*. The surprising finding of a high prevalence of antibodies to both henipaviruses and TioPV/MenPV in individual pteropid bats highlights the need for more structured studies to investigate the infection dynamics of zoonotic viruses in different bat populations across the world. It is not clear at this stage what factors are responsible for the vast difference in prevalence of antibodies to paramyxoviruses and orthoreoviruses. In the context of potential incursion of exotic bat viruses into Australia, it is noteworthy that the bat rubulavirus(es) circulating in Papua New Guinea are more MenPV-like (Australia), whereas the Papua New Guinea henipavirus(es) seem to be more NiV-like (Asia). These findings are consistent with those in Indonesia (*15*) and call for further molecular epidemiologic investigation to better assess the risk of NiV entry into Australia.

## Appendix

**Table A1:** Detection of antibodies against different viruses found in bats, Papua New Guinea, June 2008*

Location and ID no.	Species	Reactivity†					
		Hendra virus	Nipah virus	Tioman virus	Menangle virus	Nelson Bay virus‡	Broome virus
Bensbach							
2	*Dobsonia magna*					+	
4	*D. magna*					+	
8	*D. magna*		+		+		
Mabudawan							
12	*Pteropus alecto*	+	++		+		++
Madang							
13	*P. conspicillatus*	++	++	+	+		
14	*P. conspicillatus*			+	++		
15	*P. conspicillatus*	++	++			++	
16	*P. conspicillatus*	++	+				
17	*P. conspicillatus*	++	++		++		
18	*P. conspicillatus*	++	++			+	
19	*P. conspicillatus*				+		
20	*P. conspicillatus*		+		+++	+	
21	*P. conspicillatus*		++	+++			
22	*P. conspicillatus*			+++	+++	+	
23	*P. conspicillatus*	++	++	+	+++		
24	*P. conspicillatus*	++	++	+	+++		
25	*P. conspicillatus*				+		
26	*P. conspicillatus*	++	++	+++	+++		
27	*P. conspicillatus*	+	+	+	++		
28	*P. conspicillatus*	++	++	+	+		
30	*P. conspicillatus*				+++	+	
31	*P. conspicillatus*	+	+		+		
32	*P. conspicillatus*	+	+		++		
33	*P. conspicillatus*	++	++	++	+++		
34	*P. conspicillatus*			+	+++		
35	*P. conspicillatus*	+	+	+	+		
36	*P. conspicillatus*	++	++			+	
37	*P. conspicillatus*			+	+		
38	*P. conspicillatus*	+++	+++	++	+++		+
39	*P. conspicillatus*	++	++			+	
40	*P. conspicillatus*			++			
41	*P. conspicillatus*		+		+		
42	*P. conspicillatus*						
43	*P. conspicillatus*	++	++	+			
44	*P. conspicillatus*	+	+	+			
45	*P. conspicillatus*			+	+		
46	*P. conspicillatus*	++	++	++	+++		+
47	*P. conspicillatus*	+	+				
48	*P. conspicillatus*				+++		+
49	*P. conspicillatus*	+	+	+	+++	+	
50	*P. conspicillatus*				+		
53	*P. conspicillatus*	++	++	+	+	+	
54	*P. conspicillatus*	++	++		+		
55	*P. conspicillatus*	++	++	+	+++		
56	*P. conspicillatus*	+	+		+++	+	
57	*P. conspicillatus*	++	++				
58	*P. conspicillatus*	++	++	+	+		
59	*P. conspicillatus*	+	++				
60	*P. conspicillatus*			++	+		
62	*P. conspicillatus*	+	+				
63	*P. conspicillatus*	++	++				
64	*P. conspicillatus*				+++		
66	*P. conspicillatus*	+	++	+	+	+	

*No antibodies were detected in 12 of 66 bats sampled. Shading highlights samples containing antibodies to both henipaviruses and rubulaviruses. ID no., identification number of bat. †Definition of positive reactivity: for Hendra virus and Nipah virus, Virus neutralization test titer was used (+ for 10–40; ++ for 80–160; +++ for >320 or); for Tioman virus and Menangle virus, virus neutralization test titer was used (+ for 10 to 20; ++ for 40; +++ for 80 or greater); for Nelson Bay virus, ELISA reading was used (+ for OD 0.5–1.0; ++ for OD >1.0); for Broome virus, ELISA reading was used (+ for OD 0.5–1.0; ++ for OD >1.0). ‡And related viruses.
